# Investigating the Structure of Emotion: Tools, Pitfalls and Recommendations

**DOI:** 10.1007/s42761-025-00337-6

**Published:** 2025-12-15

**Authors:** Chujun Lin, Yanting Han, Umit Keles, Yue Xu, Junsong Lu, Ruoying Zheng, Ralph Adolphs

**Affiliations:** 1 Department. of Psychology, UCSD, La Jolla, USA; 2 Division of Humanities and Social Sciences, HSS 228-77, Caltech, Pasadena, 91125 USA; 3 Division of Biology and Biological Engineering, Caltech, Pasadena, USA; 4 Department of Psychology, Columbia University, 10027 New York, USA

**Keywords:** Emotion, Affect, Dimensionality reduction, Methods

## Abstract

People use hundreds of words to describe emotions and related mental states. Yet a large psychological literature and our own intuition argue for a simpler structure that underlies these attributions, since most of the words are antonyms or synonyms that map onto a few basic dimensions or categories. However, there is surprisingly little agreement on their number or interpretation: core affect is generally thought to be two-dimensional; emotion categories vary from six to over twenty; and according to some theories emotion concepts are much higher dimensional since they flexibly incorporate context. Although many of these debates originate in theoretical disagreements, we here focus on methodological sources: how stimuli are selected, tasks are chosen, and dimensionality reduction algorithms are used. We provide a survey of popular methods, their advantages and limitations, and recommendations for best practices.

Affective science studies emotion with a wealth of dependent measures, ranging from verbal ratings and self-report questionnaires to facial and bodily actions, to psychophysiological and hormonal measures and neuroimaging. There is a general consensus that emotions cause (or, depending on the theory, are in fact constituted by) most or all of these measures. Emotions are multivariate, and in general the more variables you can measure, the better. A hallmark of the field, and one shared with many other scientific disciplines, is thus the need to represent, visualize, and interpret the multivariate raw data in some simpler format – that is, some form of dimensionality reduction. In the simplest case, this could be a summary score on a questionnaire, but even there, such summary scores require careful design in the first place (for instance, from a factor analysis).

The situation is made complicated at the outset because there are hundreds, perhaps thousands, of words and concepts that we use to describe emotions, as evident in dictionaries, literatures, and social media. For example, *The Book of Human Emotions* lists 154 words across the world that describe how people feel (Smith, 2015); a classical survey of vocabulary people use to describe emotions identified 558 words with emotional connotations (Averill, 1975); and some even argue that humans can experience 34,000 emotional states situated in specific contexts (Plutchik, 1982). But a key insight from decades of research is that these numerous social attributions can be explained as the combinations of a much smaller number of dimensions. Those dimensions summarize the most important features along which emotions would differ from one another.

In this methods review, we take stock of the tools available for dimensionality reduction in affective science, and in particular the role that different approaches play in understanding the structure of emotions. The questions we discuss are the following. Are there continuous dimensions or discrete categories in the data? How many statistically meaningful dimensions are there? How interpretable are they as psychological dimensions? Although the answers to these questions are often presupposed by specific emotion theories, they depend strongly on the methods used – and there are many different methods available. Choosing the appropriate stimuli and task, deciding on the best algorithm, and ensuring its robustness and generalizability, are critical components of any empirical study. The choices made, in turn, strongly influence the evidence for or against a specific emotion theory. This means that theoretical advancement in affective science will require attention to the methods as well – they should be used not merely to accrue results consistent with a given theory, but ideally so as to help adjudicate between competing theories. We begin with an overview of some of the dimensions that are well represented in the literature – as well as some of the debates – and then provide a discussion of the methods and how they can be applied to investigate emotions.

## Psychological Spaces

We often think about and visualize data spatially. The dimensions of affective space can be thought of as a mathematical space in which different emotion states can be represented by different coordinates in the space and the relations between them can be quantified by a distance function. This has considerable advantages, since geometric tools from mathematics can be brought to bear on such representations. Importantly, a number of different coordinate systems and spatial metrics can be chosen to represent psychological relations, depending on one’s interests (Shepard, 1980). At the outset, this requires making a distinction between “dimensions” in a purely mathematical sense, as a way of summarizing data, and psychological dimensions that are ultimately thought to characterize mental representations. In general, we will follow the common assumption that statistical dimensions can (with care) often be interpreted as psychologically meaningful dimensions. But it is important to emphasize that this inference, while a common path from data to conclusions, makes assumptions and generally requires further tests to ensure validity (for example, data from neuroscience).

**Fig. 1:**
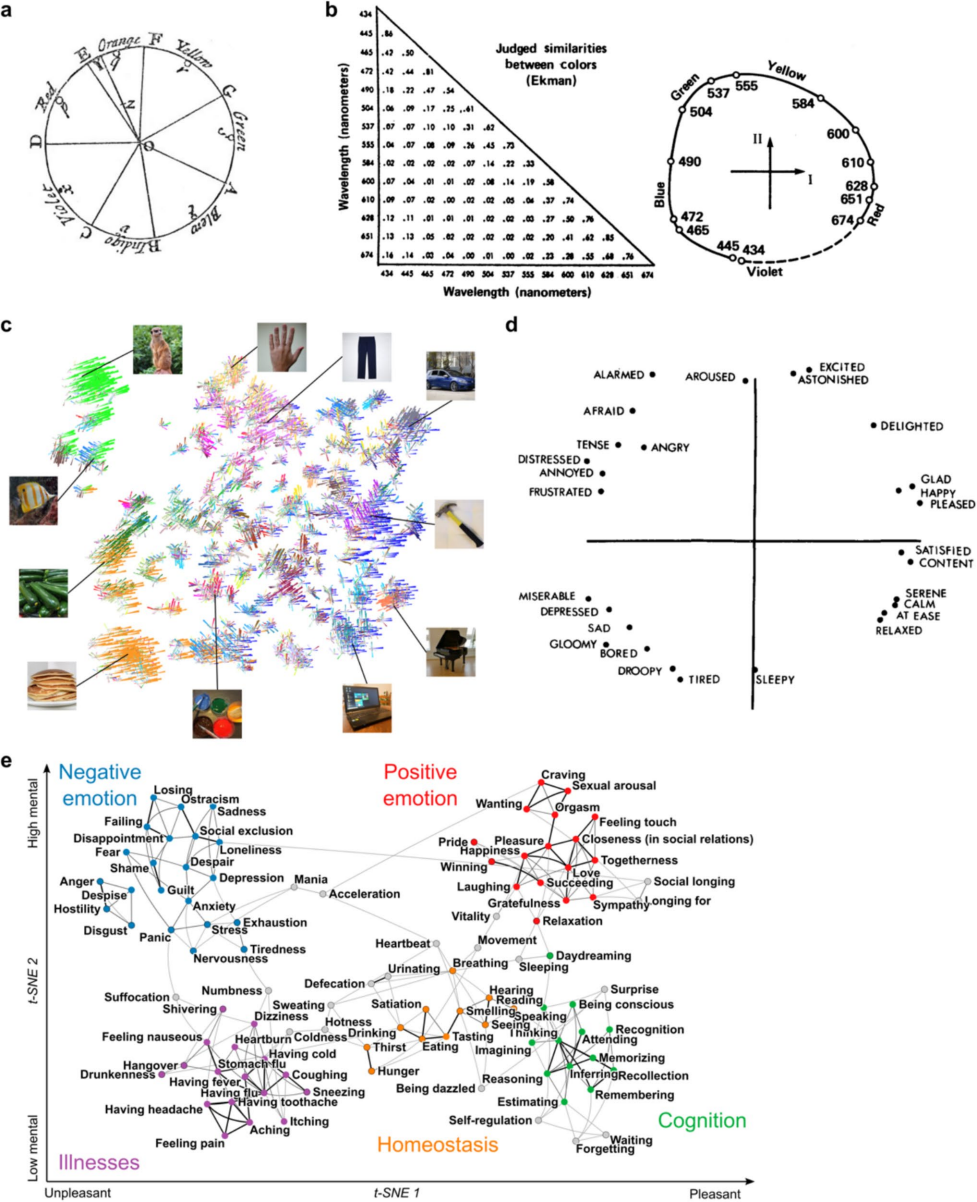
Examples of psychological dimensional spaces. **a**, **b**, Color perception. One of the earliest uses of a geometric representation of human perceptual judgments was Isaac Newton’s 1672 theory of color (Newton, 1997) (panel **a**). Based on his experiments with prisms, Newton theorized that three primary colors were sufficient to generate all the rest. Roger Shepard (Shepard, 1962) was a leader in applying dimensionality reduction methods to psychophysical data. In a re-analysis of pairwise similarity judgments of colors (left matrix, panel **b**), Shepard applied MDS to obtain a circular representation in which Euclidean distance corresponded to perceived dissimilarity in color judgments (panel **b**, figure from Gosta Ekman, 1954). **c,** Object recognition. Hebart and colleagues (2020) (Hebart et al., 2020) showed participants images of objects from a large set of nearly 2000, and used an ingenious model-based dimensionality reduction to arrive at 49 dimensions that incorporate both semantic and perceptual properties of the objects. **d, e,** Emotions. Although relatively high-dimensional spaces have been proposed for human emotions as well (Cowen & Keltner, 2017), most models propose that a low-dimensional space captures our experience as well as the semantics of emotions. The most influential model is due to (Russell, 1980) and has only two dimensions that are typically interpreted as valence and arousal, constituting core affect (panel **d**). More diverse experiences or concepts of feelings still capture most of the variance in people’s ratings in only a few dimensions (panel **e**) (Nummenmaa et al., 2018)

Some of the best studied cases of psychological spaces are perceptual, but the work has been extended into semantic and conceptual spaces as well (Fig. 1). One concrete example of perceptual spaces is human color vision, where perceived similarity of hues can be mapped onto a color wheel, in which the distance between points corresponds to psychological dissimilarity between pairs of colors (Fig. 1a, b). Three primary colors can be mixed to produce all the colors on this color wheel — the basis of RGB monitors. In this case, the psychological space was subsequently discovered to have a corresponding neurological basis: the human retina has 3 types of cones with different spectral sensitivities. From mixing the outputs of these three classes of photoreceptors, the brain can construct percepts of all the different colors humans can distinguish.

The simple example of colors can be extended to arbitrarily complex stimulus spaces, although in those cases we do not (yet) know the corresponding neural mechanism that explains the dimensions. For instance, a broad investigation of how we represent images of all kinds of objects has suggested that, just as with colors, we do so in a relatively low dimensional psychological space (Hebart et al., 2020) (Fig. 1c). Furthermore, entirely semantic spaces have been studied, as in the case of emotion words (Russell, 1980) (Fig. 1d) and broader sets of words for feelings (Nummenmaa et al., 2014) (Fig. 1e). Theoretical work on conceptual and semantic spaces has proposed a general geometric framework, where dimensions (axes) serve to define the space and convex regions within the space correspond to the concepts people use to organize and think about stimuli (Gärdenfors, 2014). This also makes the important point that not only are dimensional schemes non-unique, but they are also not at odds with clustering approaches. Psychologically similar points in a dimensional space can be assigned to a cluster, and the relationships between stimuli and clusters can be conveyed equally well by their distance in a cluster dendrogram or by their separation in a dimensional space.

We can now translate the broad picture sketched above to the problem of affective space more specifically. The stimuli are other people in the world (or states of oneself, in the case of studies of self-reported emotions). The components an observing individual has available through their senses include other people’s facial appearance, body posture, tone of voice, speech contents, and so forth (all the multimodal aspects of social perception, filtered depending on the perceiver’s own attention, engagement, and memory). The abstract challenge both in the real world and for the scientist who is studying the process in the lab is the same: How are the different cues by which we express emotions organized? That is, considering the person who is having an emotion, what mental representations cause them to produce coordinated facial expressions, alter the tone of their voice, or give us verbal reports of how they feel? And how do observers in turn construct a psychological space for representing the emotions inferred from these varied cues in their mind? In short, what are the psychological processes that allow us to represent emotions – either when we have them ourselves or when we perceive another person having them?

### The Dimensions of Emotion States

Vigorous disagreements continue about whether emotions are discrete or dimensional, and about how many categories or dimensions there should be (Barrett et al., 2018) (see Table 1). Lexical approaches (i.e., studying emotion-related words from the dictionary) have produced reliable dimensional structures, often circular manifolds in a 2-D space that are somewhat reminiscent of early theories about color vision (Fig. 1d). These two dimensions are perhaps the most agreed upon across emotion theories and are typically interpreted as valence (pleasant/unpleasant, or approach/avoidance) and arousal (or activation). According to leading theories, they constitute “core affect”, a shared dimensional space for the conscious experience of emotions from which episodes of emotion experience are constructed (Barrett et al., 2007; Russell, 1980). At the opposite extreme lie theories proposing as many as 27 dimensions of emotion experience across a variety of stimulus types (Cowen & Keltner, 2017) (see also the visualizations at alancowen.com).

**Table 1:** Examples of emotion dimensions

Reference	# Dim	First Four Dimensions	Stimuli	Task	Description	Analysis
(Ekman & Friesen, 1971)	6	(1) happiness, (2) sadness, (3) anger, (4) surprise	faces	matching (face selection)	read stories associated with one of six emotions and selected matching faces	Theory Driven
(Russell, 1980)	2	(1) pleasure-displeasure, (2) degree of arousal	words	pile sorting, rating	pile sorting based on similarity of emotion terms; self-report of current affective states	MDS, PCA
(Shaver et al., 1987)	5	fear, sadness, anger, joy, love	words	pile sorting; open-ended questions	pile sorting based on similarity of emotion terms; written accounts of emotional experiences	hierarchical clustering, MDS
(Gendron et al., 2014)	6, 3, 4	(no interpretation)	faces	pile sorting	pile sort faces based on emotion expressions	hierarchical clustering, MDS
(Cowen & Keltner, 2017)	27	(1) admiration, (2) adoration, (3) aesthetic appreciation, (4) amusement	videos	rating, free words	emotion ratings and free response labels were derived from 2,185 emotional videos	SH-CCA (split half CCA), PCA
(Nummenmaa et al., 2018)	5	(1) positive emotions, (2) negative emotions, (3) cognitive processes, (4) somatic states	words	rating, multiple arrangement	rate feeling tokens on selected dimensions and provided subjective experience similarity judgement of feelings through multiple arrangement	DBSCAN clustering, t-SNE
(Cowen et al., 2021)	16	(no explicit labels)	videos	annotation by DNN	Annotate facial expressions and contexts in 6.1 million videos from 144 countries; compute partial correlations between the context and the expression annotations across videos	CCA
(Fontaine et al., 2021)	4	(1) valence, (2) power, (3) arousal, (4) novelty	words	rating	rate emotion terms in English, French or Indonesian on 68 selected component dimensions	PCA, SCA
(Han & Adolphs, 2024)	3,4	(1) valence, (2) arousal, (3) generalizability, (4) negative affect (only for real-life)	videos, narratives, real life events	rating	ratings on 28 scales	EFA, CFA, PCA, PPCA, Autoencoder, UMAP, hierarchical clustering, K-means clustering

### A Case-Study of Methods Shaping Dimensions

Here we use the work by Cowen, Keltner and Colleagues (Cowen & Keltner, 2017) and that of our own (Han & Adolphs, 2024) to illustrate how methodological differences can lead to widely different dimensional conclusions. Cowen and Colleagues (Cowen & Keltner, 2017) first theorized that there were as many as 34 different emotions (derived from emotion taxonomies of prominent theorists), then constructed keywords for each emotion category and queried search engines and other content websites to compile a set of 2185 short videos designed to induce these emotions. Participants shown these videos were asked to provide categorical judgements on the 34 emotions about how they felt. The dimensionality reduction algorithm applied to these data was a novel split-half canonical correlation analysis (SH-CCA; taking as input the percentage of selection for each of the 34 categories).

Using a similar analysis pipeline, Cowen and colleagues also investigated emotion experiences evoked by music and artworks, as well as emotion recognition from vocal bursts, speech prosody, photos of facial-bodily expression, and facial expressions in sculptures, and similarly found a large number of dimensions (Cowen, Sauter, Tracy, & Keltner, 2019; Cown & Keltner, 2021; Keltner, Brooks, & Cowen, 2023).

**Fig. 2:**
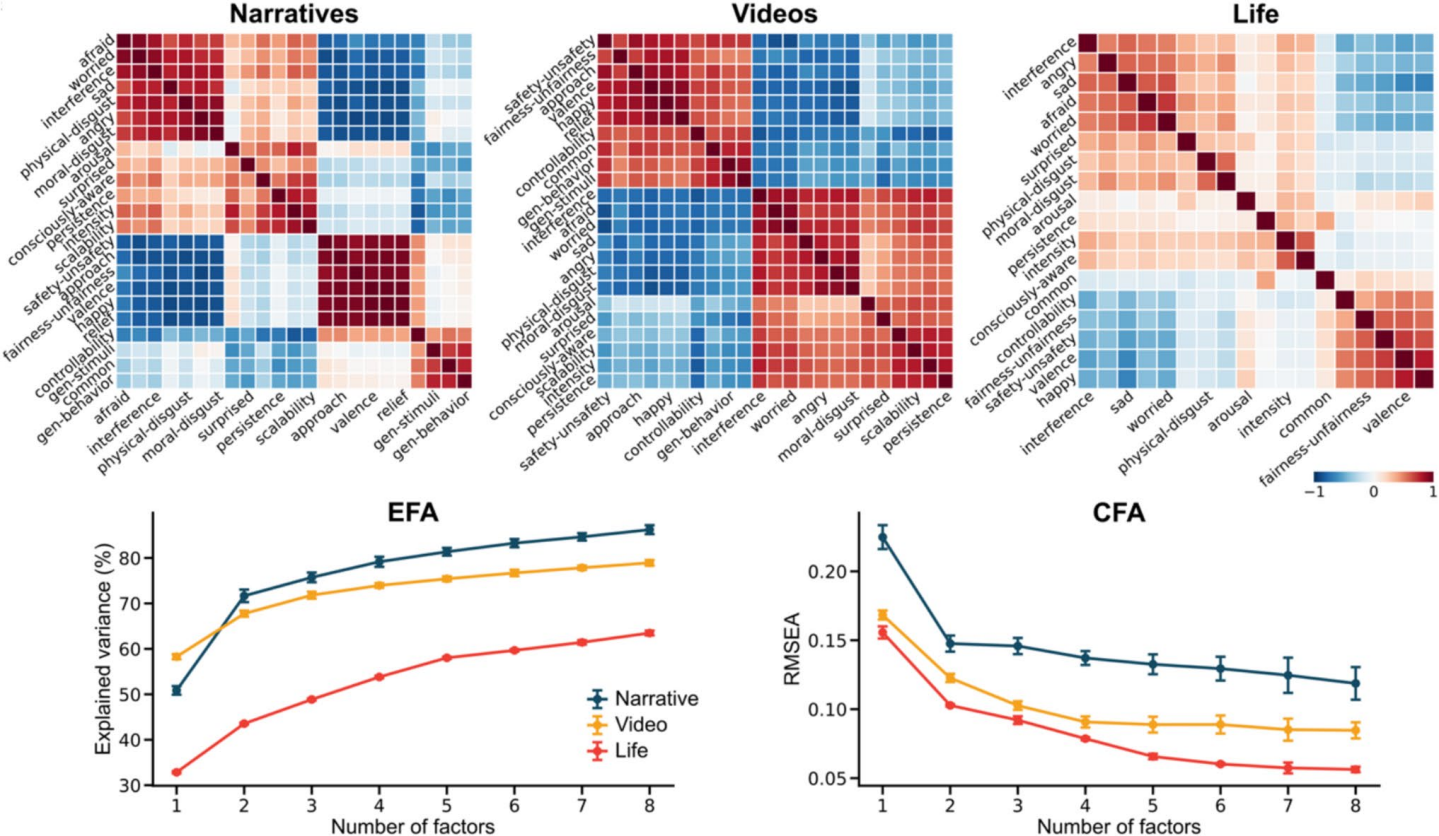
Dimensions in emotion. Shared dimensions describing emotion experiences induced by narratives, videos, or real-life events (Han & Adolphs, 2024). The top heatmaps display the correlation structure among rating terms, while the lower line graphs show results from exploratory factor analysis (EFA) and confirmatory factor analysis (CFA). Dot markers represent means, and error bars indicate standard deviations across 20 iterations of explained variance from EFA on training data (left), and the root mean square error of approximation (RMSEA) fit index from CFA on testing data (right). Visual inspection indicates a distinct change in explained variance from two to three dimensions for narratives, suggesting a 2D solution, whereas for videos and real-life-induced emotions, this trend is less pronounced. The authors employed a cross-validation approach to empirically determine the dimensionality. Adapted with permission from (Han & Adolphs, 2024)

However, Barrett et al., (2018) have suggested that the SH-CCA method would favor recovering how well the rating covariances conform to prespecified emotion categories, rather than uncovering latent psychological dimensions. A different result comes from our own work, (Han & Adolphs, 2024), which also used the identical video stimuli (as well as additional types of emotion-inducing stimuli, narratives and real-life experience), and collected ratings on a diverse range of scales (28 in our study). Although the exact rating words we used differed from Cowen & Keltner’s, the sheer diversity of words used would seem to ensure that both studies would uncover a similar number of emotion dimensions. Whereas Cowen and Keltner (2017) concluded that there were 27 emotion dimensions, our study concluded there were 3 (valence, arousal, and a novel dimension of “generalizability” in the case of emotions evoked by videos) (Fig. 2). How could this be?

A large part of the answer, we believe, lies in the different methods for dimensionality reduction used, and the different metrics used to determine the number of dimensions. In our study (Han & Adolphs, 2024), we used a number of common dimensionality reduction methods (cross-validated exploratory factor analysis and principal component analysis, among others) that all generated similar results. The input to these algorithms were continuous ratings participants gave about their emotion experience. The primary metrics we used to decide on the number of dimensions/factors were cross-validation (EFA cross validated with CFA) and the proportion of variance explained (the metric that most studies use). We concluded from this that we had found a relatively low-dimensional psychological space for emotion experience, since our 2–3 dimensions accounted for most of the variance in participants’ ratings. By contrast, Cowen and Keltner (2017) used categorical judgments of the emotion, a very different dimensionality reduction method, and statistical significance rather than explained variance as the metric for deciding how many dimensions to keep. Even if the additional variance explained is miniscule, Cowen & Keltner’s metric of statistical reliability would differentiate emotions for which people have distinct concepts. Both approaches are defensible, but we suggest that whereas ours can be interpreted to yield dimensions that account for most of the variance in participants’ emotion experience, Cowen & Keltner’s can be interpreted to yield dimensions that account for statistically reliable discrimination between people’s concepts for emotions.

### More on Discrepant Dimensions

How choices of stimuli, tasks, and dimensionality reduction algorithms shape the discovered dimensions of a psychological space is also evident in other psychological spaces beyond emotions. The literature provides a bewildering plethora of dimensions by which people organize concepts and perceptions of traits (e.g., two dimensions of agency and communion (Abele & Wojciszke, 2007)), mental states (e.g., three dimensions of rationality, social impact, and valence (Thornton & Tamir, 2020)), actions (e.g., six dimensions of action (Thornton & Tamir, 2022)), and relations (e.g., five dimensions of relationship concepts (Cheng et al., 2023)). The variety of different frameworks clearly stems from the different methods and criteria used for dimensionality reduction, but they also depend on the heterogeneous social phenomena that are being studied.

These examples highlight a deeper cause for discrepant emotion dimensions in the literature. The phenomena to be explained are just different in many cases. Paul Ekman’s six or so categorical emotions (Ekman, 1993) are based on the production and recognition of social signals from facial expressions, and thus are specifically about social communication. Jim Russell’s two-dimensional circumplex model of emotions (Russell, 1980) is based on the meanings of words, and thus about the semantics of language (to be fair, Russell’s original paper did include one experiment in which he also compared his circumplex model to rated emotion experiences, so it is not as though the model is irrelevant for experience). Cowen and Keltner’s 27 dimensions (Cowen & Keltner, 2017) are based on categorical decisions about the emotions experienced from the videos (and, subsequent to that study, also many other types of stimuli), asking participants to map their judged emotion onto pre-determined emotion categories. Nummenmaa’s maps of subjective feelings are again based on the meanings of words (Nummenmaa et al., 2018), and his bodily maps of emotions are based on people’s concepts of where in the body they would hypothetically feel an emotion if they had it (Nummenmaa et al., 2014). We could go on, but hope the point is clear: these are all different phenomena! In addition to the methodological distinctions, it is critical to ensure that what is mapped into the dimensional space corresponds to what it is that the dimensions are supposed to represent – that is, all theories and studies need first and last to tackle validity (Box 1).

**Table:** 

Box 1: Validity
What one can conclude about dimensions of emotion depends on what one sets out to investigate, so it is essential to clearly state the goals and limitations of a study. Many disagreements in the literature can be attributed to ambiguity in the phenomenon under investigation. The following questions should be considered
***1. Ground truth.*** Is the study investigating the objective emotion of a human or animal, or is it investigating how a human observer represents (or anthropomorphizes) these? Analysis of videos of behavior using an unsupervised algorithm would investigate the former; analysis of people’s ratings of the behavior would investigate the latter; and analysis using a machine-learning model that has been previously trained to classify behavior (that is, a supervised model) may be a mixture depending on biases in the model’s original training
***2. Experience or concept.*** A very common conflation in the literature is whether a study is investigating emotion experience (e.g., the conscious feelings induced by watching charged videos) or people’s concepts for those states (e.g., asking people to rate words that describe emotions). Unless an effort is made to disentangle these, most studies offer an unknown mixture of these two (e.g., reading brief narratives depicting emotional events may produce emotion ratings on the basis of what participants think the correct emotion concept should be, and/or on the basis of the emotion actually induced in participants as they imagine the story; individual differences in the relative weights of these are also likely)
***3. Language or not.*** The most common tasks involve ratings of stimuli using a set of words chosen by the experimenter, which immediately limits the dimensions that can be discovered and also requires some conceptualization (cf. 2 above). Dependent measures that do not require language include sorting stimuli, or behavioral responses to stimuli other than language, or brain imaging data. However, in non-aphasic adult humans, language and concepts may inevitably occur (that is, people will always think about the stimuli, or their own states, using concepts and language, and people will always have in mind the demands of the experiment). Studies in animals or comparisons across cultures can be helpful here
***4. Qualitative vs. quantitative.*** Some studies may not be intended to make any claims about psychological dimensions (e.g., normative ratings or weights on attributes provided in a database of stimuli for the purpose of convenient stimulus selection). Some methods are also intended for visualization rather than strong analytic claims (e.g., t-SNE or UMAP). By contrast, some studies are intended to generate or test a psychological theory. A study should clearly state whether its conclusions are intended to be qualitative or quantitative
***5. Application.*** A study should state its intended application, which generally also entails stating limitations on generalizability. Is it intended to provide exploratory qualitative results? To quantitatively test hypotheses about stimuli, tasks, participant individual differences or psychological theories? To provide an explanation of a phenomenon, or to provide predictions that could be diagnostically useful for clinical questions? It is common to pay lip service to “ecological validity,” but we feel that this is misguided: the study should just be clear on what its intended goal is and should then ensure that the design of the experiment is valid with respect to that goal. The goal need not be to understand the real world (whatever exactly that means)

## Methodological Challenges and Recommendations

**Fig. 3:**
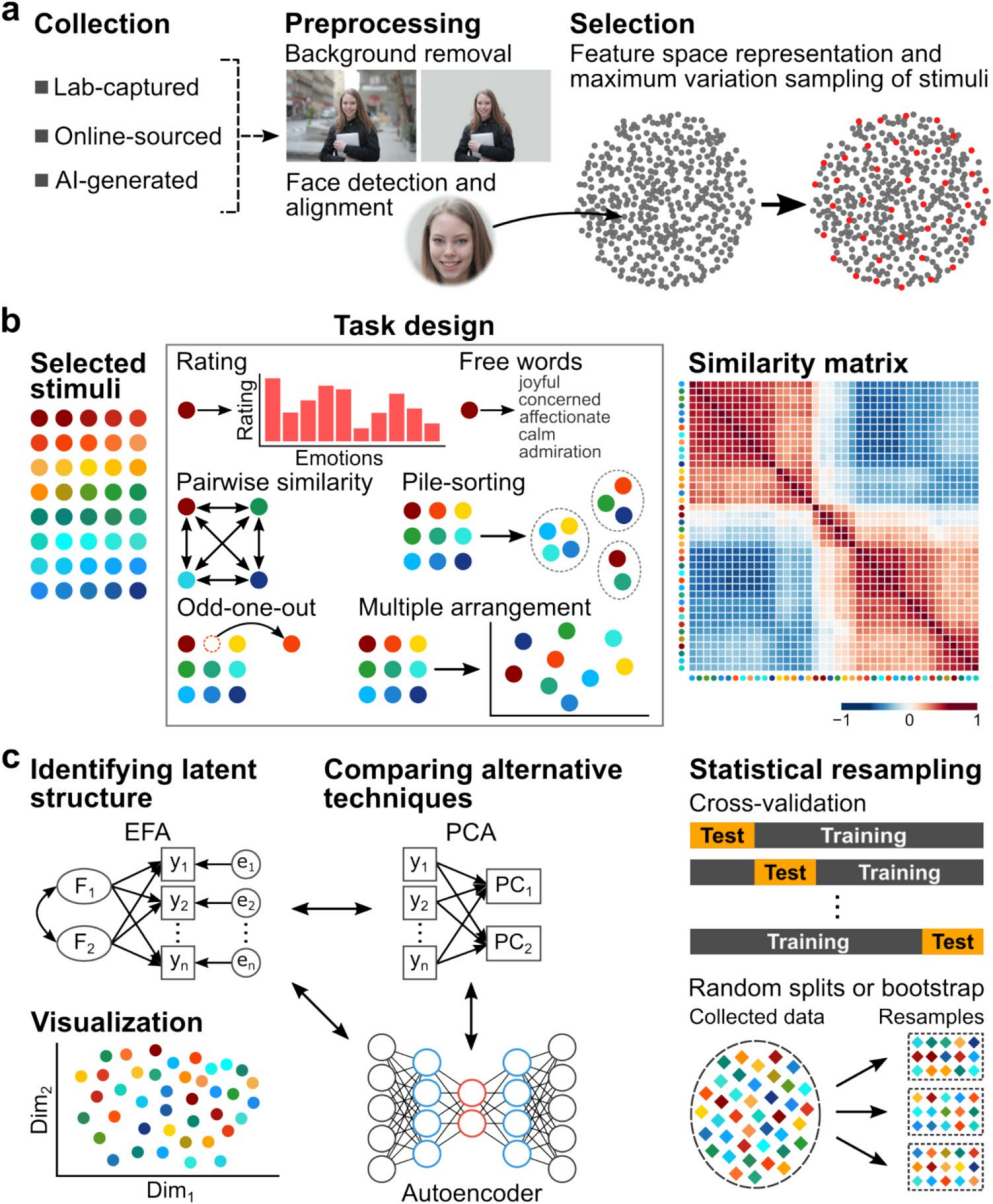
Schematic of a study. **a**, Assembling stimuli requires attention to validity (what phenomenon is studied; Box 1), generalizability (bias, completeness), and statistical power (range, variability). Dimensions can only be revealed if the stimuli vary with respect to them. **b**, Task and data collection. Tasks make different psychological demands, and have different efficiency and reliance on language. Construction of a (dis-)similarity matrix is often done by aggregating sparse individual data across participants and/or sessions, but this raises the question of whether the psychological space ultimately modeled applies to group or individual performance. **c**, As with the tasks, multiple dimensionality reduction algorithms are available. Ideally, a study uses more than one type of stimulus set, more than one type of task, and more than one type of dimensionality reduction algorithm to obtain convergent results. Data-driven approaches using resampling can help estimate the dimensionality of the model solution in an unbiased way

**Fig. 4:**
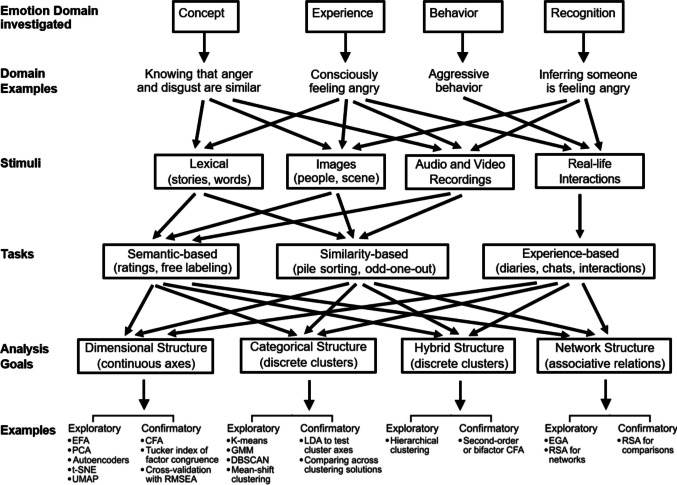
Methodological considerations for investigating the dimensions of emotion. Starting from the domain of research, this flowchart guides decisions about stimulus selection, task selection, and analysis strategies. The final section organizes analytic strategies based on assumptions about latent structure—dimensional, categorical, hierarchical/hybrid, or network-based—and distinguishes between exploratory from confirmatory approaches. Examples of commonly used methods are listed under each analytic pathway. While structured as a flowchart, this figure is not a strict decision tree: many research paths may converge on similar analytic approaches, reflecting the fact that emotion research often relies on shared methods across different theoretical aims and task designs. The chart is best interpreted as a modular guide to core methodological options, rather than a prescriptive linear pipeline

The dimensions discovered in a study depend on the stimuli chosen, the task, and the algorithms for data analysis (Fig. 3). At each step of the study, the investigator needs to consider the overarching goals, and the best way to design the experiment (Fig. 4) so that the dimensions revealed are valid with respect to the phenomenon putatively studied, are as complete and robust as possible, and generalize (**Box 1**).

### Stimuli

There are a number of stimulus databases that already provide partly validated stimuli, including affective words (ANEW (Bradley & Lang, 1999)), images (affective images, IAPS (Lang et al., 2005); faces (Chelnokova et al., 2014; DeBruine & Jones, 2017; Ma et al., 2015), full body images (Connor et al., 2021; Kosti et al., 2019; Zhou et al., 2021), scene images (Xiao et al., 2010)), audios (Cowen, Elfenbein et al., 2019; Cowen, Laukka et al., 2019, p. 24), narratives (Skerry & Saxe, 2015), short videos (Cowen & Keltner, 2017; Ong et al., 2021) or longer movies (Chen & Whitney, 2019) as well as music (*DEAM Dataset—Database for Emotional Analysis of Music*, n.d.). Furthermore, there are some collections of multiple datasets that can provide a useful and updated overview (e.g., the KAPODI database of Diconne et al., 2022). Some of these are more suitable for inducing emotions in the participant (e.g., emotional videos), others more for making judgments about the emotions and mental states of another person (e.g., faces), and yet others simply probe concepts and semantics (e.g., single words). While most databases only include stimuli generated in laboratory conditions (e.g., posed photos of participants), a growing number include stimuli that are more naturalistic (Bainbridge et al., 2013; Biel & Gatica-Perez, 2013; Liu et al., 2015). With the development of user-friendly tools for scraping contents from the internet, researchers can now assemble a large variety of stimuli from social media posts, news reports, profile photos, home-made videos, and other sources. An important consideration here is privacy and copyright, issues that become even more acute if any of the stimuli are to be shared or used in figures of publications (Merriman, 2014).

In addition to selecting and scraping stimuli by the researchers one can also prompt human participants to provide stimuli (e.g., surveying which major social groups to include for uncovering stereotyping dimensions (Koch et al., 2016)), and synthesize stimuli de novo from computational models (e.g., artificial faces and bodies (Hu et al., 2018; Peterson et al., 2022) or using modern AI tools (Lu et al., 2025)). These provides a convenient source of unlimited stimuli with fewer privacy and copyright considerations but require justification and validation to ensure that synthetic stimuli engage the same processes as do real ones, and have known biases.

Regardless of how stimuli are sourced or generated, one initially ends up with far more than can feasibly be rated by participants in a study. Similarly for rating scales (i.e., emotion labels on which to collect ratings) – even if one decides to select a list of emotion words from prior research, these are generally too numerous to use completely (Peabody, 1987; Saucier & Goldberg, 1996). Subsampling stimuli and rating scales is typically done in three ways. First, large sets of stimuli and ratings are in fact feasible, if they are allocated sparsely across a large sample of participants so that every participant does not need to rate every stimulus (Han & Adolphs, 2024; Jones & Kramer, 2021), although in that case one needs to be careful in extrapolating findings from the group aggregate data to individual psychology. Second, researchers often have theoretical reasons to choose only a subset of stimuli, for instance those that correspond to dimensions in their theory (thus possibly biasing the study). Third, the most common data-driven approach to sub-sampling is simple random selection. But there is a better approach.

A goal of sub-sampling should be to generate a representatively distributed set of stimuli or rating scales, and random sampling will simply reproduce biases already present in the original set. It is thus generally advisable to explicitly consider the generalizability of interest to the study, and to sub-sample the stimuli accordingly (Brunswik, 1947). Maximum variation sampling will ensure that the full range of variability is sampled along chosen features (Lin et al., 2021, 2022; Lu & Lin, 2024) (Fig. 3a); more specific criteria such as equating males and females, or races, need to be combined with specific exclusionary criteria to produce the final stimulus set of interest. Maximum variation sampling consists of two steps: first, extracting the features of interest (what is the domain of generalization that is desired?), and second, selecting a smaller number of stimuli that maximizes their range along those features. Accessible automated tools now exist for characterizing words (He et al., 2021; Wang & Lin, 2024), facial structural and dynamic features (Lin et al., 2023; Ren et al., 2022), body shape and poses (Kocabas et al., 2021; Zheng & Lin, 2024), and gist of scenes (Gong et al., 2018). Once the features of interest in the stimuli are quantified, we can then compute the distances (in that feature space) between all stimuli in the available set and select a subset (with a known subset size, ideally determined based on a power analysis) that maximizes the sum of distances between the stimuli (Lin et al., 2021, 2022). Maximum variation sampling can be further applied together with stratified sampling of different categories (e.g., female faces and male faces), generating distinct subsets of stimuli that differ maximally along the features of interest within the given category.

### Tasks

Figure 3b schematizes some of the tasks used and Table 2 provides example studies. There are two superordinate types: one directly maps the stimuli into a set of high-dimensional coordinates within a psychological space whose dimensions are under investigation, the other measures the relation between pairs of stimuli without assigning them any specific coordinates within the psychological space. Perhaps the most common task for the first type involves rating the selected stimuli on a list of attributes (e.g., rating the perceived emotion of a face stimulus on a set of words using 7-point Likert scales). Once attention has been given to the scope of the study and its generalizability (e.g., selecting the rating words and the face stimuli), a rating task produces a set of coordinates as a vector of ratings on the list of attributes (i.e., a high-dimensional coordinate) for each stimulus, permitting the derivation of a similarity metric across stimuli (e.g., by correlating the rating vectors) that can be used as an input to a dimensionality reduction algorithm. For instance, in Fig. 2, participants rated their emotion experience on all the words shown in the figure. The strength of the correlation in the resulting similarity matrix (top heatmaps in Fig. 2) already shows that there are two broad groups of scales: those scales whose higher ratings indicate more negative valence, and those scales whose higher ratings indicate more positive valence. This impression was then quantified with factor analysis (Fig. 2, bottom panels).

**Table 2:** Examples of tasks. Selected examples illustrating the use of specific tasks, their strengths and limitations

Methods	Use Cases	Descriptions	Strengths	Limitations
Rating	(Han & Adolphs, 2024)	Using a Likert scale to rate the pre-selected items (e.g., stimuli) on an interval scale	Highly standardized procedure and higher reliability; easy to analyze	Limited scope and suffers from selection bias; potentially inflated associations between emotions caused by forced response
Checklist	(Asch, 1946)	Select words from a list that are most in accordance with the given items (e.g., stimuli)	Higher comparability between conditions or participants; easy to analyze	Limited scope and potential selection bias; less suitable for sophisticated analysis
Free words	(Cowen & Keltner, 2017)	Write words that naturally come to mind while seeing the experimental stimuli	Rich content; less constrained	No clear standard to determine the granularity of dimensions; higher variability in responses; more difficult to analyze
Pairwise similarity	(Mijin, Wager, Phillips, 2022)	Uses a Likert scale to rate the similarity between two stimuli	Directly surveying similarity	No control over judgment context; potential shift in judgment over trials; inefficient
Odd-one-out	(Dima et al., 2023)	Pick the odd stimulus from a subset of stimuli	Forced choice, context provided by the set of stimuli compared together and thus lower possibility of shift in judgment over trials	Inefficient (for a workaround see (Hebart et al., 2020))
Pile-sorting	(Gendron et al., 2014)	Sort stimuli into N piles (predetermined or self-determined)	Forced categorical decisions, rich similarity judgment context (provided by the entire set of stimulus being sorted together); fewer trials needed to cover all stimuli	Less constrained than odd-one-out, and thus potentially biased by each participant’s sorting strategy
Multiple arrangement	(Xu, Keles, Lin, & Adolphs, 2025)	Arrange stimuli in 2D so that the distance between each stimulus pair represents their similarity	Continuous and rich similarity judgment; (sets of stimuli arranged together); fewer trials needed to cover all stimuli; commercial Meadows platform available for task	Less constrained than odd-one-out, and thus potentially biased by each participant’s arranging process

The tasks listed here represent popular methods used in studies investigating the dimensions of emotions and other psychological attributes. They are not intended to be exhaustive. The use cases are examples that are not exhaustive either

Other task examples that provide dimension coordinates for stimuli include the checklist method (Asch, 1946) and the unconstrained word generation method (Cowen & Keltner, 2017), which both map stimuli into a high-dimensional space based on the occurrence of words. Whereas the checklist method asks participants to select from a list of words provided by the experimenters, the unconstrained word generation method lets participants freely generate their own words. Both tasks produce a set of words that can be converted to a high-dimensional vector of word occurrence (a vector of 0 s and 1 s across all unique words), permitting the derivation of a similarity metric across stimuli (e.g., by correlating the occurrence vectors). To avoid bias in choosing lists of words to begin with, a recommended approach is to combine the rating task or the checklist task with the unconstrained word generation task (Lin et al., 2021).

One might think that it would be best to dispense with words altogether and to obtain direct similarity judgments on the stimuli. Such approaches have the benefit of not being constrained by the rating scales, and perhaps not even requiring language. Some example tasks include pairwise similarity (Koch et al., 2016) (participants directly rate similarity between pairs of stimuli, requiring many comparisons if complete), the odd-one-out task (Dima et al., 2023) (participants judge which stimulus differs the most from others in a set), the pile sorting task (Gendron et al., 2014) (e.g., participants freely sort all stimuli into multiple piles that indicate similarity, which can be more efficient if done coarsely), and the multiple arrangement task (Dobs et al., 2019) (e.g., participants spatially arrange multiple stimuli at once to indicate how similar or different the stimuli are in terms of a certain topic or certain attributes). All these tasks produce a (direct or derived) similarity matrix across the stimuli (e.g., faces), which can then be used as input to dimensionality reduction algorithms such as multidimensional scaling, which projects the stimuli into a space of N dimensions such that the psychological similarity corresponds to the Euclidean distances in the N-dimensional space.

However, despite the benefits of minimizing the conceptual constraints of rating scales, all these latter types of tasks that directly measure relations between stimuli face a number of challenges. First, they are often inefficient: methods like pairwise similarity and odd-one-out necessitate a substantial number of trials for complete sampling. Second, the similarity matrices generated describe the stimuli (rather than the rating scales) and thus depend critically on selecting the stimuli and often produce spaces of indeterminate or uninterpretable dimensions (especially if no explicit attributes are ever used in the task). Another problem is that it is psychologically challenging to get participants to do what you want them to do: pairwise similarity judgments, odd-one-out judgments, matching, pile sorting, and multiple arrangement all require participants to choose some features or attributes of the stimuli on the basis of which to perform the similarity judgment. Without further instruction (such as that provided by explicit rating scales) it is unclear what attribute individual participants are using. Thus, when using tasks that directly measure similarity among stimuli, it is critical to give clear instructions and validate that the measures collected reflect what the investigators intend to measure.

### Analyses

Dimensionality reduction is a major and rapidly growing field that applies to data across scientific disciplines. There is a bewildering array of algorithms: univariate, multivariate, linear, nonlinear, supervised, unsupervised (Table 3), each with particular advantages and assumptions (Nguyen & Holmes, 2019). Broad aims range from simplification and visualization in order to facilitate qualitative interpretation of complex data (e.g., visualizing multivariate patterns using t-SNE or UMAP (Cowen, Elfenbein et al., 2019; Cowen, Laukka et al., 2019; Han & Adolphs, 2024)), to preprocessing and denoising of data for subsequent analysis (e.g., using ICA to remove motion artifacts in fMRI data), or construction of models for prediction and explanation of the psychological space (often with EFA (Lin et al., 2021) or PCA (B. C. Jones, 2021)).

**Table 3:** Examples of dimensionality reduction algorithms. Selected examples illustrating the use of specific algorithms, and their strengths and limitations

Methods	Use Cases	Descriptions	Strengths	Limitations
Principal components analysis (PCA)	(Han & Adolphs, 2024)	Map high-dimensional data into a lower-dimensional space by finding a number of uncorrelated components, which are linear combinations of observed variables, that account for as much variation in observed data as possible	Computationally less intensive; estimates parsimonious representations of the observed variables while preserving variance	No theoretical claims about the underlying data structure; treats all variance as meaningful without modeling error variance; deciding the number of components can be subjective; common approach does not address nonlinear relationships underlying the data
Exploratory factor analysis (EFA)	(Han & Adolphs, 2024)	Assumes that observed variables are correlated because they are influenced by shared latent factors. Observed variables correspond to coordinates in the latent axis space	Identified factors are often interpretable; accommodates the measurement error of observed variables; allows and models the correlation between factors	No universally acknowledged standard for determining the optimal number of factors; common approach does not model potential nonlinearity
Confirmatory factor analysis (CFA)	(Han & Adolphs, 2024)	Tests theories. Observed variables are represented as combinations of latent factors based on prior assumptions. Theoretical assumptions are evaluated using various goodness-of-fit indices	Enables a theory-driven approach; models can be tailored to specific questions and are highly flexible	Does not allow for the discovery of new factor structures; assumes linear relationships
Hierarchical clustering	(Han & Adolphs, 2024)	A clustering method that builds a hierarchy of clusters. Observations are merged based on some similarity metric, resulting in a nested structure	Highly flexible, does not require specification of the number of clusters (unlike k-means clustering)	Sensitive to outliers and lacks a unifying criterion for splitting the hierarchy into clusters
UMAP & t-SNE	(Cowen & Keltner, 2017); (Cowen, Elfenbein et al., 2019; Cowen, Laukka et al., 2019)	Nonlinear dimensionality reduction methods that embed high-dimensional data for visualization in a low-dimensional space	Capable of handling nonlinear relationships between observations. UMAP captures both local and global structures	Both require careful optimization of hyperparameters and can be subjective. t-SNE is computationally demanding and sometimes struggles to capture the global structure
Exploratory graph analysis (EGA)	(Truhan et al., 2021)	Assumes that observed variables form clusters within a network that represent the partial correlation between variables. The clusters consist of densely connected variables and represent underlying dimensions	Automatically estimates the number of dimensions; higher accuracy in identifying the number of dimensions compared to traditional methods (e.g., parallel analysis)	Items that are multidimensional or have poor stability can lead to unstable dimensional structures
Louvain Algorithm	(Zhang et al., 2019)	Optimizes an objective function (e.g., modularity) to partition the network into communities where nodes within the same community are more densely connected than to the rest of the network	Efficient and scalable for large networks	May produce disconnected communities; suffers from the resolution limit, causing small communities to be merged into larger ones
Multidimensional scaling (MDS)	(Xu, Keles, Lin, & Adolphs, 2025)	Projects data into lower dimensions by converting dissimilarities among data points into spatial distances. Particularly useful for visualizing patterns in the relationships among stimuli	Highly versatile, applicable to any type of similarity or dissimilarity data. Supports both metric (actual distances) and non-metric (rank order) methods, efficiently manages linear and nonlinear interactions	Sensitive to the choice of distance metric and scaling strategy, affecting result stability and interpretability. Highly susceptible to noise and does not scale well with large datasets
Canonical correlation analysis (CCA)	(Cowen & Keltner, 2017)	Reduces dimensions by identifying pairs of new dimensions that best capture correlations between two datasets. It finds linear combinations of original variables in each set that are maximally correlated with each other, ensuring each new dimension is orthogonal to the previous ones in its set	Provides insights into how sets of variables from different datasets are interrelated, highlighting the most informative features that explain these relationships	Requires large sample sizes to ensure stable and reliable results. Assumes linear relationships between the variables of both datasets, limiting its ability to capture complex, nonlinear interactions. Alternatives include partial least squares regression and reduced rank regression
Non-negative matrix factorization (NMF)	(Dima et al., 2023)	Reduces dimensions by decomposing a non-negative data matrix into two lower-dimensional non-negative matrices, capturing inherent patterns and extracting features that contribute most significantly to the data structure	Enforces non-negativity, which simplifies interpretation by providing a parts-based representation where each dimension can be directly associated with meaningful features of the input data	Sensitive to initialization and the number of components, affecting the stability and reproducibility of results. Assumes data can be approximated by additive combinations of a limited number of components
Simultaneous Component Analysis (SCA)	(Fontaine et al., 2021)	Extracts shared latent components from multiple related data matrices. It finds a set of linear combinations that have the same component structure across all datasets by modeling all matrices simultaneously. These components maximize the explanation of variance across datasets	Enables joint analysis of multiple related datasets while preserving their shared structure; facilitates direct comparisons across groups or conditions by extracting components with common loadings	The same component structure is assumed to make sense in all datasets; similar to PCA, it assumes a linear relationship and does not explicitly model noise or error variance
Autoencoder	(Thornton, Rmus, Vyas, Tamir, 2023)	An artificial neural network designed to learn a lower-dimensional representation of input data through an encoder that maps inputs to a hidden layer and a decoder that reconstructs them from the hidden layer. Training minimizes the reconstruction loss, often using a mean squared error variant	Highly adaptive and can handle various types of data distributions and non-linear interactions within data	Requires large datasets and substantial computational resources. Prone to overfitting, especially if the network architecture is too complex relative to the amount of data

The algorithms listed here represent popular methods used in studies investigating the dimensions of emotions and other psychological attributes. They are not intended to be exhaustive. The use cases are examples and are not exhaustive either

Focusing on the aim of obtaining a psychological space, three broad considerations are critical when selecting dimensionality reduction methods: first, whether one wishes to explore categories/clusters, or continuous dimensions; second, whether one wishes to test a specific dimensional theory or instead to discover dimensions using a data-driven approach; and third, whether the data one has are dimensional coordinates of the stimuli (e.g., from a rating task) or just the relations among the stimuli (e.g., from a pile sorting task). One general recommendation is to use several methods and test that they converge on the same conclusions.

If one wishes to discover continuous dimensions using a data-driven approach, the two most common dimensionality reduction methods in psychology are exploratory factor analysis (EFA) and principal components analyses (PCA). Both EFA and PCA can work for data that provide specific coordinates of the stimuli (e.g., data measured from a rating task, checklist task, or unconstrained word generation task). The biggest difference between EFA and PCA is that EFA assumes two sources of variance: that shared among all variables (e.g., each of the emotion words with their ratings across the stimuli) due to a common mechanism (i.e., driven by a common set of dimensions), and that unique to each variable and not shared with other variables (e.g., measurement errors that are unique to each variable). By contrast, PCA only assumes the first (shared) source of variance. Both EFA and PCA are linear methods (linear combinations of the factors or components obtained explain the variance in the observed data). However, PCA produces component scores that are linear combinations of the observed variables weighted by eigenvectors, while EFA views observed variables as linear combinations of the underlying factors. The dimensions are orthogonal (uncorrelated) when obtained from PCA, but not necessarily when obtained from EFA. A popular method for relational data (data measured from the pairwise similarity task, the odd-one-out task, the matching task, the pile sorting task, or the multiple arrangement task) is multidimensional scaling (MDS), which has a number of versions available (Rosenberg et al., 1968). Finally, with the availability both of larger datasets and of computational resources, nonlinear methods using neural networks provide advanced flexibility. For instance, autoencoders encode high-dimensional data to a low-dimensional bottleneck from which the data can be reconstructed (Lin et al., 2021; M. A. Thornton et al., 2023).

After discovering dimensions, one may wish to compare the results with existing theory. One popular method to follow up EFA is confirmatory factor analysis (CFA) (Brambilla et al., 2011), which specifies how each variable is generated based on a set of underlying hypothesized dimensions. A common misconception about CFA is that it is simply the confirmatory equivalent of EFA. While EFA assumes that all variables have non-zero weights on all dimensions, CFA typically assumes that only a certain subset of variables has weights on a certain subset of the dimensions (to more explicitly test different hypotheses or theories about how different variables are generated by different dimensions). To most meaningfully compare the dimensions obtained in one study with those from another, we recommend using the Tucker index of factor congruence (Goldberg, 1990; Lin et al., 2021).

While most analyses of emotion structure are conducted at the group level, this approach may obscure meaningful heterogeneity in how individuals represent emotions. Idiographic and multilevel methods address this by estimating both within- and between-person factor structures, allowing researchers to examine whether the same dimensions emerge across individuals and how strongly they are expressed. Multilevel factor analysis (MLFA) and multilevel structural equation modeling (ML-SEM) extend EFA/CFA by modeling individual-level (within-person) and group-level (between-person) structures simultaneously (Roesch et al., 2010; Hox et al., 2017). For instance, a two-level model may include level-1 loadings that capture each participant’s idiosyncratic emotion space, while level-2 parameters reflect the group-average structure and its variability. Representational similarity analysis (RSA) offers a non-parametric alternative: by computing pairwise dissimilarities among emotion stimuli separately for each participant and comparing these matrices to group-level or theoretical models, RSA quantifies convergence and divergence in representational geometry (Kriegeskorte et al., 2008). Another emerging approach is idiographic network analysis, which estimates person-specific Gaussian graphical models—where nodes are emotion features and edges are partial correlations—to reveal each individual’s unique network architecture (Mansueto et al., 2023). Together, these methods provide a nuanced view of how emotional dimensions, clusters, or networks emerge and vary across individuals.

### Using Data-Driven Criteria

Dimensionality reduction by itself does not yet determine the number of dimensions/clusters into which the data should be fit. Both for choosing the number of dimensions, and for quantifying the robustness of the final solution, data-driven approaches are now feasible and are generally recommended over other approaches (Fig. 3c). The total number of dimensions that will fit all variability in a dataset will equal the number of stimuli or rating scales. The function that describes the residual variance explained as further dimensions are added is generally a monotonically decreasing curve, and the task is to decide when further variance explanation becomes meaningless. Several different metrics are available, based on the proportion of variance itself, an error metric, or a statistic, depending on the dimensionality reduction method used (Hastie et al., 2009; Johnson and Wichern, 2007; Lorenzo-Seva et al., 2011).

The elbow method, conceptually identical to the scree plot, plots either eigenvalues (in PCA/EFA) or within-cluster variance (in k-means) against the number of components or clusters, and identifies a point of diminishing returns. While intuitive, this method is inherently subjective when the “elbow” is ambiguous. The Kaiser criterion retains components with eigenvalues above unity, on the rationale that each explains at least one variable’s worth of variance, but often overestimates dimensionality when variables are numerous or communalities are low. Permutation-based approaches, such as parallel analysis, generate null distributions from randomized data and retain components or clusters whose explanatory power exceeds chance. These methods are statistically principled and help control overfitting, though they can be computationally intensive. Fit-index criteria (e.g., RMSEA in EFA, stress cutoffs in MDS, silhouette scores in clustering) offer clear decision thresholds and help balance false positives and negatives, though their performance can vary with sample size, model complexity, and estimation method. Information criteria such as AIC and BIC embed dimensionality selection within a likelihood framework that penalizes complexity to mitigate overfitting. Cross-validation techniques—including split-half stability, predictive accuracy on held-out data, or reconstruction error—provide a robust, empirical basis for evaluating the generalizability of both low-dimensional embeddings and cluster solutions. Velicer’s Minimum Average Partial (MAP) test minimizes average squared partial correlations in orthogonal decompositions and is useful when communalities are moderate. The Very Simple Structure (VSS) index evaluates how well simplified loading or distance-reconstruction matrices approximate observed relationships, emphasizing interpretability alongside fit, while the Hull method identifies a kink on the convex hull of fit-versus-complexity curves, offering a general-purpose rule across factor models, MDS stress analyses, and clustering validity metrics. While some criteria (e.g., RMSEA) are specific to particular model classes, others—permutation tests, cross-validation, and stability indices—apply broadly across linear, non-linear, and graph-based approaches. Because each method embodies different statistical assumptions, sensitivities, and interpretability considerations, no single criterion is universally optimal. We therefore recommend triangulating across multiple approaches, weighing both empirical evidence and theoretical coherence when determining the number of dimensions or clusters in emotion-related data.

Another data-driven method that could be used both to compare dimensionality reduction results and to compare the original data structure without any dimensionality reduction is Representational Similarity Analysis (RSA) (Kriegeskorte et al., 2008). For the former, one might use the same set of stimuli but with different tasks or dimensionality reduction methods to obtain two different dimensional spaces. To compare them, one could use the weights across stimuli on each of the dimensions to construct a similarity matrix between stimuli, and then compare the similarity matrices across the two sets of dimensional solutions. For the latter, one might compare different psychological spaces with the similarity space from a brain imaging study (Freeman et al., 2018; Skerry & Saxe, 2015; Xie et al., 2021) directly without reducing these high-dimensional measures to a low-dimensional space first. The method essentially involves a second-order correlation (usually one with the least assumptions, such as a Kendall-Tau correlation) performed on pairs of similarity matrices. The similarity matrices with the highest correlation are then interpreted as the most related.

If one wishes to discover discrete categories/clusters of the psychological space using a data-driven approach, the two main classes of clustering methods are parametric and nonparametric. Two popular parametric methods are K-means clustering (Lin & Thornton, 2023) (which assumes clusters are spherical and works by repeatedly assigning data points to a nearest cluster centroid) and Gaussian Mixture modeling (GMM) (Ernst et al., 2021) (which assumes that data is generated from a mixture of Gaussian distribution and works by iteratively updating their mean and variance so as to maximize the probability that the observed data was generated by the model). The popular nonparametric examples are hierarchical clustering (Han & Adolphs, 2024) (which works by iteratively merging the closest pairs of clusters or splitting a single cluster into smaller clusters based on the distances between data points) and mean-shift clustering (Jones & Kramer, 2021) (which works by iteratively shifting candidate cluster centroids towards the mean of the data points within their neighborhood until the shifts become negligible). To compare discrete clusters with continuous dimensions/factors, Jones and Kramer used linear discriminant analysis (LDA) to construct a “cluster axis” (that best separated the discovered two clusters) which was then compared to EFA factors and PCA components (Jones & Kramer, 2021).

Another important approach is offered by network-based community detection methods, which offer a flexible, data-driven alternative to classical factor-analytic and clustering approaches for uncovering the latent structure of emotion. These methods operate on graphs or similarity matrices derived from co-occurrence, rating similarity, or perceptual similarity data, and aim to identify clusters of densely interconnected elements. For instance, Infomap (Rosvall & Bergstrom, 2008) applies principles from information theory to find clusters that minimize the description length of a random walker's path through the network, making it especially suitable for identifying hierarchical and overlapping structures. The Louvain method (Blondel et al., 2008) iteratively maximizes modularity—a measure of within-cluster edge density compared to chance—and aggregates modules to reveal hierarchical community structure. The Walktrap algorithm (Pons & Latapy, 2005) similarly uses random walks, clustering nodes based on the probability of co-visitation within short walks, thus producing a hierarchical dendrogram from which nested clusters can be extracted. In contrast, stochastic block models (SBMs; Holland et al., 1983) provide a generative, probabilistic framework, modeling the likelihood of connections based on latent community membership and supporting compact, interpretable representations akin to latent‐class solutions. Although these methods have seen limited use in affective science to date, they are well suited to applications involving similarity graphs derived from multiple arrangement tasks, pairwise similarity judgments, or language-based embeddings. For example, a recent study by Sacchi and Dan-Glauser (2024) applied hierarchical community detection to appraisal–response networks and uncovered a three-module architecture of emotion components, demonstrating the potential of these tools to reveal latent structure from complex relational data.

Finally, data-driven criteria can also be applied to help interpret the dimension solutions. In general, dimensionality reduction methods do not provide interpreted dimensions or clusters (i.e., the analysis does not tell investigators what each dimension means or what each cluster means), which is left up to the intuitions of the investigators. However, data-driven approaches for interpreting the dimensions based on factor loadings are now available. For instance, large language models can be used to compute the semantic distance between each attribute and a potential label of a dimension, and then one can compare these distances with the factor loadings of those attributes on the target dimension, finally selecting the label (or a set of labels) that most highly correlate with the target dimension.

## Summary and Future Directions

There are a wealth of stimuli and selection criteria, tasks, and algorithms that psychologists have available to study the structure of emotion (see Fig. 4 for some of the options). We can summarize some key findings from this field so far. First, the number of dimensions that describe emotions vary substantially across studies. This variability is not unique to the field of affective science, as it is also observed for studies investigating other psychological spaces, such as mental states and personality traits. Second, across different studies, the first few dimensions that account for most of the variance usually overlap, often corresponding to valence and arousal (core affect). This pattern is again seen also for other psychological spaces, such as warmth and competence for personality variation and trait perception – while there is debate about how many dimensions are meaningful, there is usually convergence on the first few dimensions that account for the bulk of the variance in a study. And third: the reasons for the disagreements are multiple. In many cases, investigators are simply addressing different questions (such as the experience of one’s own emotion, the inference of another person’s emotion, or the semantic meaning of emotion words; Box 1). The other major reason is that different methods can be used in every step of the investigation pipeline: the dimensions will depend on the stimuli selected, the task used, and finally the dimensionality reduction algorithm and data-driven criteria applied (Table 2, Table 3, Figs. 3, 4).

We began this review by noting the distinction between “dimensions” as merely statistical properties of the data, versus features of mental representations. In the strongest sense, the dimensions that scientists discover correspond to psychologically real dimensions by which people represent other minds (or their own). The implication is that the phenomenology of our conscious experience of emotions also occurs in such a space — how we think and feel about another person or about ourselves can be characterized in this psychological space. Another implication is that all cognition and behavior that is based on the representation of the other person depends on a subsequent read-out from this dimensional space. The geometry of the representation and the details of the read-out should matter to cognition and behavior. These are currently hot topics in both machine learning (Cross & Ramsey, 2021) and cognitive neuroscience (Ho et al., 2022; Nieh et al., 2021), where representations need to be factorized or disentangled in order to permit generalizations that abstract across stimuli (e.g., representing people who physically look very different as all having a similar emotion).

Finally, one could hypothesize that the psychological dimensions of emotion are not merely useful for guiding adaptive behavior, but that they have actually converged on fundamental dimensions in the social world that are objectively “real”. This “realness” can be interpreted in two different perspectives. One is that these dimensions may align with the dimensions that are actually encoded in the biological brain and neurons (Bao et al., 2020; Chang & Tsao, 2017; She et al., 2024) (just as the three color dimensions are encoded by the photoreceptors, Fig. 1). Second, just as there are physically real structures and symmetry operations in the world (Higgins et al., 2022), there may be objective structure in the behaviors and experiences of humans and animals that can similarly be summarized through investigating the dimensions of emotion.

“Possibly we can aspire to a science of [the] mind that, by virtue of the evolutionary internalization of universal regularities in the world, partakes of some of the mathematical elegance and generality of theories of that world. The principles that have been most deeply internalized may reflect quite abstract features of the world, based as much (or possibly more) in geometry, probability, and group theory, as in specific, physical facts about concrete, material objects.” – Roger Shepard, BBS 2001(Shepard, 2001).
